# Development of an intravascular ultrasound elastography based on a dual-element transducer

**DOI:** 10.1098/rsos.180138

**Published:** 2018-04-25

**Authors:** Cho-Chiang Shih, Pei-Yu Chen, Teng Ma, Qifa Zhou, K. Kirk Shung, Chih-Chung Huang

**Affiliations:** 1 Department of Biomedical Engineering, National Cheng Kung University, Tainan, Taiwan; 2 Department of Biomedical Engineering, University of Southern California, Los Angeles, CA, USA; 3 Institute of Biomedical and Health Engineering, Shenzhen Institutes of Advanced Technology, Chinese Academy of Sciences, Shenzhen, China; 4 Medical Device Innovation Center, National Cheng Kung University, Tainan, Taiwan

**Keywords:** intravascular ultrasound, acoustic radiation force, shear wave elasticity imaging, plaque, atherosclerosis

## Abstract

The ability to measure the elastic properties of plaques and vessels would be useful in clinical diagnoses, particularly for detecting a vulnerable plaque. This study demonstrates the feasibility of the combination of intravascular ultrasound (IVUS) and acoustic radiation force elasticity imaging for detecting the distribution of stiffness within atherosclerotic arteries *ex vivo*. A dual-frequency IVUS transducer with two elements was used to induce the propagation of the shear wave (by the 8.5 MHz pushing element) which could be simultaneously monitored by the 31 MHz imaging element. The wave-amplitude image and the wave-velocity image were reconstructed by measuring the peak displacement and wave velocity of shear wave propagation, respectively. System performance was verified using gelatin phantoms. The phantom results demonstrate that the stiffness differences of shear modulus of 1.6 kPa can be distinguished through the wave-amplitude and wave-velocity images. The stiffness distributions of the atherosclerotic aorta from a rabbit were obtained, for which the values of peak displacement and the shear wave velocity were 3.7 ± 1.2 µm and 0.38 ± 0.19 m s^−1^ for the lipid-rich plaques, and 1.0 ± 0.2 µm and 3.45 ± 0.45 m s^−1^ for the arterial walls, respectively. These results indicate that IVUS elasticity imaging can be used to distinguish the elastic properties of plaques and vessels.

## 1. Introduction

Atherosclerosis is a slowly progressive and systemic disease characterized by the accumulation of plaque through the deposition of calcium, fibrin, cholesterol, fat and other substances in the intima of large and medium-sized arteries, including the aorta, coronary artery and peripheral arteries [1–3]. The specific disease that involves atherosclerotic plaques building up on the walls of the coronary artery is coronary artery disease (CAD), which is the most common type of cardiovascular disease that is the leading cause of death globally [4]. The rupture of a vulnerable atherosclerotic plaque, which is composed of a thin fibrous cap and an underlying necrotic lipid core, is a major contributor to acute cardiovascular events [3,5]. Several studies have revealed that such a lipid-rich plaque may rupture and break away from the vessel wall when it cannot withstand the stress imposed by the pulsatile pressure of the blood [6]. Plaque vulnerability is a complex and multifactorial process that relates to both morphological and mechanical characteristics [7]. The accurate geometric features of plaque and surrounding vessel wall have been well presented by current imaging modalities. Therefore, the challenge to predict plaque ruptures would depend on the precise knowledge of the mechanical properties of the arterial wall and plaque [8]. The ability to quantitatively estimate the geometrical and mechanical properties of the plaque and arterial vessel wall would be useful in clinical diagnoses because it would allow the physician to determine the degree of arteriosclerosis and estimate the risk of plaque rupture [9,10].

Intravascular ultrasound (IVUS) is a commonly used standard modality for the intravascular assessment of coronary atherosclerotic disease and for guiding interventional therapies such as stent implantation. Based on the echogenicity of acoustic waves, IVUS can allow cross-sectional visualization of the coronary artery wall and provide quantitative lumen dimensions and plaque area. However, the sensitivity and specificity of IVUS for detecting the composition of a plaque are poor due to the minimal contrast between different types of soft tissue [11]. With the aim of solving this problem, several investigators have reported different multimodality imaging techniques that combine different optical methods for plaque characterization. However, none of these methods provides information about the mechanical properties of a plaque, such as IVUS with photoacoustic imaging [12], IVUS with optical coherence tomography [13] and IVUS with near-infrared spectroscopy [14].

Over the past two decades, various ultrasound techniques have been proposed for assessing the mechanical properties of soft tissues. In 1991, Ophir *et al*. developed an imaging technique called elastography to measure the elastic properties of soft tissue based on applying an external force to the tissue and measuring its deformation at different depths. This method can be used to reconstruct the stiffness distributions within the tissue according to its internal strain profile along the transducer axis [15]. Several groups have applied this approach for IVUS purposes in so-called IVUS elastography. This technique involves inducing different strains in the plaque and epicardial vessel by the application of intraluminal pressures [16–18]. However, in order to avoid the detection of different tissues, the imaging frames in intravascular elastography can only be acquired near end-diastole, which involves a pressure difference of 4–5 mmHg as determined using elastograms. This produces slight errors in the pressure measurements that would cause considerable errors in estimations of elastic properties [19].

In addition to the elastography methods involving the application of direct forces, acoustic radiation force (ARF) elasticity imaging—which involves applying an ARF in order to remotely palpate tissue—has been developed by researchers as an alternative to conventional ultrasound elastography [20]. ARF elasticity imaging involves applying a short-duration (less than 1 ms) impulsive ARF to the tissue, whose mechanical properties can then be determined directly from the induced dynamic motions of the tissue. ARF elasticity imaging techniques fall into two general categories: (i) acoustic radiation force impulse (ARFI) imaging, which is used to measure the localized displacement within the region of excitation (ROE) caused by the transfer of momentum from the longitudinal wave [21], and (ii) shear wave elasticity imaging (SWEI), which is used to measure the deformation from the shear wave that propagates transversely from the ROE [22]. Preliminary studies have demonstrated the feasibility of using ARFI imaging for atherosclerosis and plaque characterization [21,23–26]. ARFI imaging can be used to distinguish various components of plaques, such as calcium, collagen deposits, lipid pools and fibrous caps [27,28]. In addition, SWEI techniques have been used for assessing the mechanical properties of arterial walls, especially in the carotid artery [29,30] and aorta [31], but only a few studies have used it for detecting atherosclerosis [32]. Most of the above-mentioned studies involving ARFI and SWEI have investigated the carotid and popliteal arteries. The low resolutions of these imaging systems due to the relatively low ultrasound frequency employed (typically 3–7 MHz) make it difficult to use non-invasive elastography to estimate the mechanical properties of plaques in smaller arteries, such as the coronary artery, which is particularly important for CAD.

High-frequency ultrasound (generally 30–50 MHz for the coronary artery) provides a better spatial resolution, but the associated higher acoustic attenuation reduces the penetration of the radiation force into the tissues. Meanwhile, using lower-frequency ultrasound (generally below 10 MHz) for generating the ARF cannot provide sufficient resolution for imaging the coronary arteries. This has led to proposals to use a dual-frequency transducer in high-resolution ARFI imaging for assessing the elastic properties of tissues [33,34]. Those studies used lower-frequency ultrasound to induce the tissue displacement and then higher-frequency ultrasound to track the tissue deformation in ARFI imaging. Even though the dual-frequency transducer used was too large for IVUS imaging, the intensities of ARF needed to induce sufficient displacements in several arteries of pigs and artificial lipid-rich plaques have been reported [34]. On the other hand, this concept of ARFI-IVUS imaging is now feasible for CAD diagnosis due to the ability to fabricate miniaturized transducers that can be inserted into the artery [35,36]. Dual-frequency IVUS transducers were fabricated with two co-aligned beams. Although those studies did not yield any biological data, the experimental results did demonstrate the feasibility of ARFI-IVUS imaging using a miniaturized dual-frequency transducer. The ARFI-IVUS technique has gradually been developed for the purpose of detecting atherosclerosis and seems useful in providing the relative elastogram for visualizing the location of plaques and thereby aid clinical diagnoses. However, the ability to quantify the mechanical properties of the plaque and arterial vessel wall is still important for the clinician to evaluate the risk of rupture of a vulnerable plaque. The ARFI technique can only provide the relative stiffness because of the unknown force. To our knowledge, the SWEI method, which is one of the useful techniques for quantifying the elastic properties of soft tissue, has not been applied to IVUS imaging.

The aim of this study was to combine ARF elasticity imaging and the IVUS technique for assessing the mechanical properties in atherosclerosis. In this study, a dual-frequency IVUS transducer with 8.5 and 31 MHz elements was fabricated: the 8.5 MHz element was used to generate the ARF that induces the propagation of shear wave, while the 31 MHz element was used to detect the induced dynamic displacements of the tissues. The system performance was verified in tissue-mimicking gelatin-based phantoms. *Ex vivo* experiments were carried out using a rabbit abdomen aorta containing lipid-diet-induced plaques. The obtained experimental results show the feasibility of using ARF-IVUS elasticity imaging for distinguishing atherosclerosis in a clinical diagnosis.

## 2. Material and methods

### 2.1. Dual-frequency intravascular ultrasound transducer

**Figure 1.**
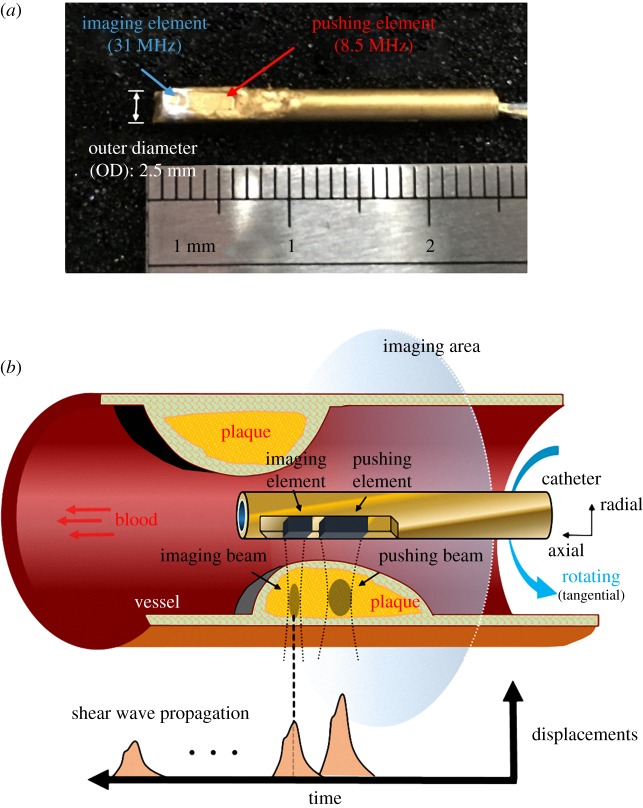
Scanning scheme of the ARF-IVUS elasticity imaging system. Photograph of the dual-frequency IVUS transducer (*a*). Scanning method of the transducer inside the artery (*b*).

Figure 1*a* shows the dual-frequency transducer with two unfocused elements which was fabricated in this study (NIH Ultrasonic Transducer Resource Center, University of Southern California, Los Angeles, CA, USA). The outer diameter of the transducer is 2.5 mm, and the active areas of the pushing and imaging elements are 2 × 3 mm^2^ and 1 × 1 mm^2^, respectively. Pb(Mg1/3Nb2/3)O3-PbTiO3 (PMN-PT) and Pb(Zr,Ti)O_3_ (PZT) was used in this study to fabricate the 31 MHz element and 8.5 MHz element, respectively. The pitch width between the pushing and imaging elements is 0.5 mm. The 8.5 MHz pushing element was used to generate the ARF that stimulated the propagation of the shear wave, and the induced dynamic tissue displacement was detected by the 31 MHz imaging element for reconstructing the image, as shown in figure 1*b*.

### 2.2. Experimental set-up

**Figure 2.**
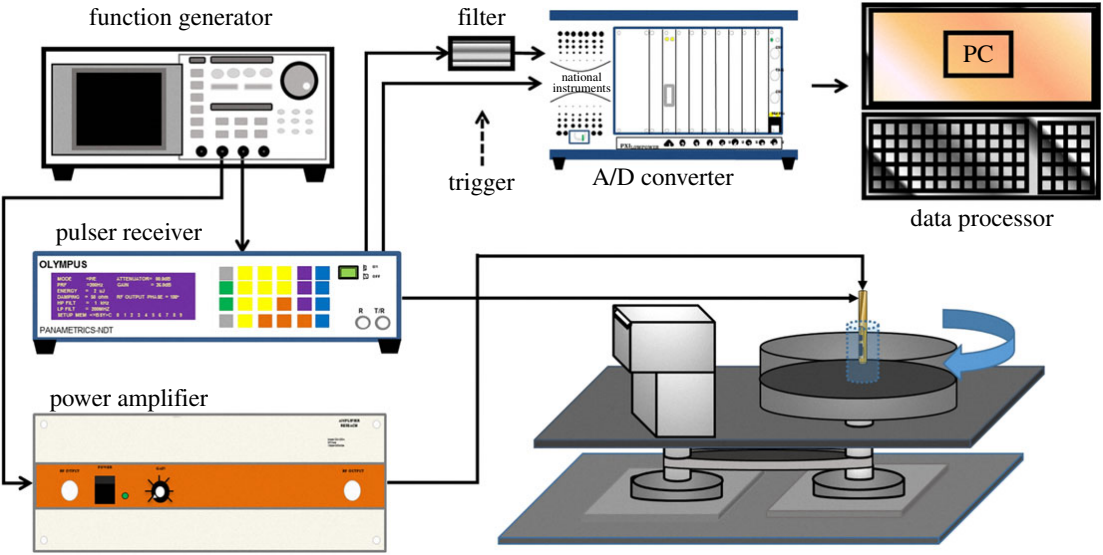
Block diagram of the experimental system.

The experimental system is illustrated in figure 2. The pushing element was excited by a function generator (AFG3252, Tektronix, Beaverton, OR, USA) connected to an RF power amplifier (25A250, Amplifier Research, Souderton, PA, USA). Sinusoidal 8.5 MHz tone bursts with durations from 0.2 to 1 ms were provided by the function generator (corresponding to 1700 to 8500 sinusoidal cycles). The peak-to-peak amplitude of the output tone burst from the power amplifier ranged from 85 to 170 V. The 31 MHz element of the dual-frequency transducer was used in imaging to detect the localized displacement of the tissue. A pulser–receiver (5900PR, Panametrics, Waltham, MA, USA) with a bandwidth of 200 MHz was used to drive the imaging element of the transducer for transmitting and receiving ultrasound signals with a pulse repetition frequency (PRF) of 20 kHz. The received ultrasound signals that had been backscattered from the tissue were amplified and filtered using a built-in variable-gain amplifier and a 30 MHz bandpass filter (BBP-30+, Mini-Circuits, Brooklyn, NY, USA). The pulser–receiver was triggered by the function generator. The PRF trigger of the function generator was also used to synchronize the acquisition of the backscattered signals at a maximum sampling frequency of 2 GHz by an 8-bit analogue-to-digital converter (ADC) (PXI-5152, National Instruments, Austin, TX, USA). The pulser–receiver, function generator and ADC were synchronized using a trigger from the function generator while the imaging and pushing elements were operating continuously.

**Figure 3.**
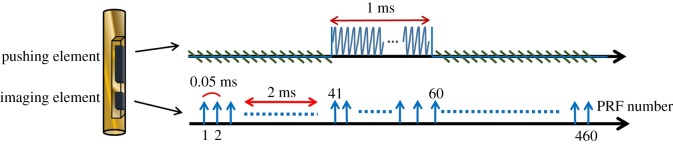
Representative timing diagram for the experiments. Pushing pulses produced by the 8.5 MHz element were synchronized (with a 2 ms delay) with the initial imaging pulses of the 31 MHz element.

The dual-frequency transducer was fixed on the stage and the sample was placed at the centre of the rotating motor platform to allow scanning at different angles. A two-dimensional (2D) IVUS image was obtained by moving the platform through 250 steps per revolution, with 460 A-lines being acquired at each angle. A representative timing diagram for the two elements is shown in figure 3. The ARF was applied by the pushing element at 2 ms after the imaging element started to receive the signals backscattered from the tissue. However, the imaging element was affected largely by the pushing element as two elements were activated simultaneously (the received echo signals were coupled with the pushing burst). To avoid the interferences, the displacement estimations were performed on the echo signals after the excitation period of ARF in each representative timing step. The signals before the excitation period of ARF were used as the references for determining the initial position of the tissue. The tissue displacement at each detectable position was measured as a function of time using a cross-correlation tracking algorithm under a sliding window of 77 µm with 50% overlap [37]. The tissue displacements induced by shear wave propagation along the axial direction of vessel were measured at different positions, as shown in figure 1*b*. The wave-amplitude images were reconstructed by the peak displacements of shear wave amplitudes; while the wave-velocity images were reconstructed by the shear wave velocities. In the study, the shear wave velocities (*c*_s_) were simply estimated by the ratio of the distance between pushing and imaging element (*d*) and time-to-peak (*t*_peak_) as follows:

2.1 $$c_{s}=\frac{d}{t_{\mathrm{peak}}}.$$

The peak displacement and the velocity were assumed to be zero when the average backscattered signals in the correlation window were smaller than a certain threshold (0.01 Vpp) for distinguishing the vessel wall from the lumen with saline solution. Data analyses were performed on a personal computer using Matlab (The MathWorks, Natick, MA, USA).

### 2.3. Gelatin phantom

**Figure 4.**
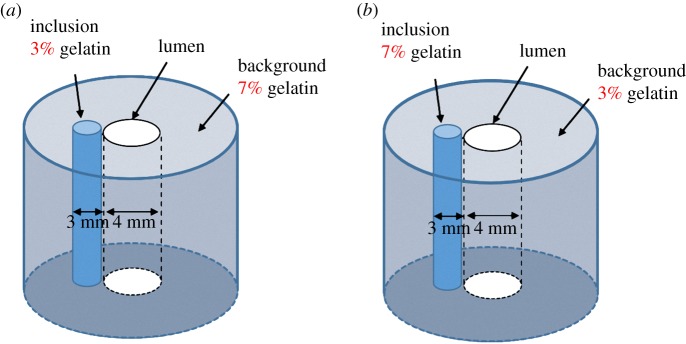
Sketches of the two types of gelatin phantom: type I, containing the softer rod (3% gelatin) (*a*); and type II, containing the harder rod (7% gelatin) (*b*).

The system performance was verified by constructing tissue-mimicking phantoms using gelatin (type A, Sigma-Aldrich, St Louis, MO, USA) at different concentrations to simulate soft tissues of various stiffnesses. The same concentration (3%) of graphite powder (Sigma-Aldrich) was added to all gelatin phantoms as particles for producing backscattering. Two types of phantom comprising gelatin at concentrations of 3% and 7% were used in this study. The shear moduli of 3% and 7% gelatin were around 0.45 and 1.65 kPa, respectively [38,39]. Diagrams of the gelatin phantoms are shown in figure 4. Phantom type I was manufactured by embedding a 3%-gelatin rod into a 7%-gelatin background, and phantom type II was manufactured by embedding a 7%-gelatin rod into a 3%-gelatin background. Each of these phantoms had a 4 mm lumen in the centre, and the diameter of the embedded rods was 3 mm. The diameters of the gelatin backgrounds are all 35 mm. Because the stiffness of the gelatin phantoms could be affected by the temperature, all of the experiments were performed at a constant room temperature of 25°C [40].

### 2.4. Aorta samples

Rabbit abdomen aorta was used in the *ex vivo* experiment in the study. Aorta atherosclerosis was induced in a male New Zealand White rabbit by applying a combination of a cholesterol-rich diet (containing 1.5% cholesterol) and FeCl_3_-induced injury to the arterial walls [41]. One week after commencing a cholesterol-rich diet, the rabbit was anaesthetized intraperitoneally with ketamine (35 mg kg^−1^), xylazine (5 mg kg^−1^) and acepromazine (0.75 mg kg^−1^). Anaesthesia was maintained during the procedure with isoflurane inhalation via a face mask. The rabbit was then placed in a dorsal recumbent position, and a midline abdomen incision was made to surgically expose the aorta that was then carefully dissected out. The arterial wall was then injured by placing a strip of filter paper (0.5 × 2.0 mm^2^) saturated in 5% FeCl_3_ solution onto the adventitia of the mid portion of the artery for 5 min. The filter paper was then removed, and the incision was sutured closed. The rabbit was sacrificed after 8 weeks, and then tissue samples of aortic atherosclerosis were obtained.

The aorta was cut to a length of 8 mm, which included the atherosclerotic portion. A tube of diameter 2.6 mm was carefully inserted into the lumen of the aorta and attached to the centre of the bottom of a plastic circular container with a size of 40 mm (diameter) × 10 mm (height). A 9%-gelatin solution was poured into the container out of the aorta and then the container was kept at 5°C for about 20 min for gelatin coagulation. After that, the tube was pulled out carefully from the aorta. The container with the aorta sample was placed on the rotating platform and saline solution was injected into the lumen of the aorta. The experiments were performed by inserting the IVUS transducer into the lumen of the aorta at a constant room temperature of 25°C.

## 3. Results

**Figure 5.**
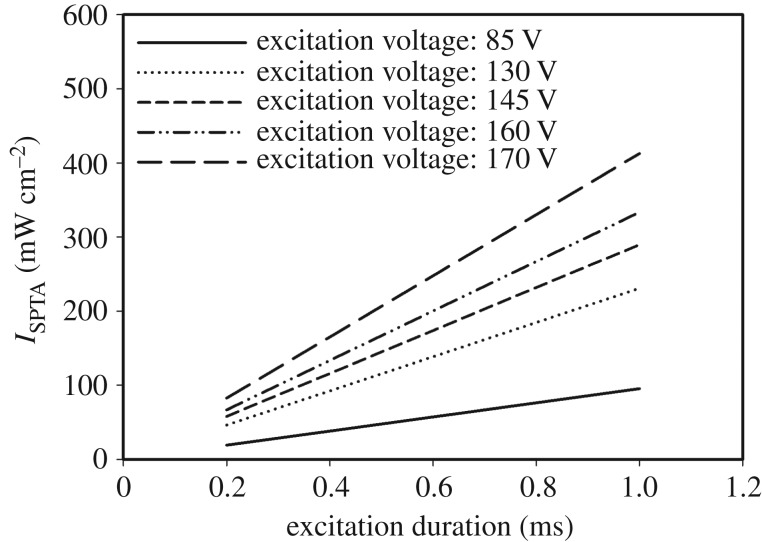
Acoustic intensities (*I*_SPTA_ values) generated by the 8.5 MHz element measured by a hydrophone for different excitation voltages (85, 130, 145, 160 and 170 V) and for excitation durations from 0.2 to 1.0 ms with a step of 0.2 ms.

The intensity of ARF of the pushing element was measured by a calibrated hydrophone (HNP-0200, Onda, Sunnyvale, CA, USA), which was carried out by following our previous study in detail [34]. The measured spatial peak temporal average intensity (*I*_SPTA_) values of the excitation duration of the pushing element at various voltages are plotted in figure 5. The acoustic intensity increased linearly with the excitation duration and voltage. The maximum and minimum *I*_SPTA_ values were 412.9 and 19.0 mW cm^−2^ for excitation voltages of 170 and 85 V with excitation durations of 1.0 and 0.2 ms, respectively.

**Figure 6.**
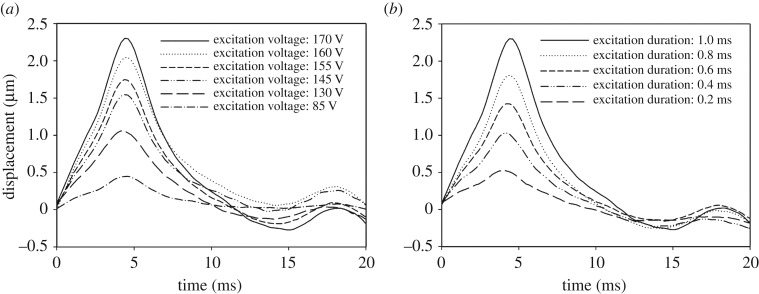
Dynamic displacement curves measured in a 3%-gelatin phantom for excitation voltages from 85 to 170 V at a fixed excitation duration of 1 ms (*a*) and for excitation durations from 0.2 to 1 ms at a fixed excitation voltage of 170 V (*b*).

Advanced studies for developing an ARF-IVUS elasticity imaging system need to determine the optimal excitation parameters (including input voltage and duration) for exciting the transducer when generating the ARF. The system performance was verified by using the phantoms constructed from the gelatin at concentrations of 3% and 7%. Figure 6 shows the particle displacements induced by the pushing element in a homogeneous 3%-gelatin phantom. The excitation voltage applied to the pushing element was varied from 85 to 170 V with the tone-burst duration kept fixed at 1.0 ms. The peak displacements were measured from 0.4 to 2.4 µm, with *t*_peak_ ranging from 4.3 to 4.4 ms, as shown in figure 6*a*. The peak displacement increased with the excitation voltage, while the times at which the peak occurred were almost identical in all cases. In a second series of experiments, the excitation duration of the ARF was varied from 0.2 to 1.0 ms with a fixed excitation voltage of 170 V. Both the maximum displacement and the time to reach it increased with the excitation duration, as shown in figure 6*b*. The peak displacements were measured from 0.5 to 2.3 µm with *t*_peak_ ranging from 3.8 to 4.4 ms, as shown in figure 6*a*.

**Figure 7.**
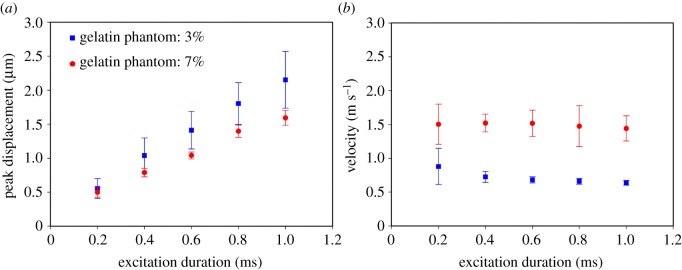
Peak displacement (*a*) and velocity (*b*) of the shear wave propagation in the 3%- and 7%-gelatin phantoms for excitation durations from 0.2 to 1 ms at a fixed excitation voltage of 170 V. Data are mean and s.d. values.

It is expected that a higher ARF will induce greater displacements, and figure 6*a*,*b* shows that the peak displacements increased with the excitation time in the 3%-gelatin phantom. Figure 7 further demonstrates the peak displacement and the velocity induced by the ARF under excitation durations from 0.2 to 1 ms with a fixed excitation voltage of 170 V in the 3%- and 7%-gelatin phantoms, respectively (as measured in four samples for each phantom type). Data are mean ± standard deviation. The peak displacements were measured from 0.6 ± 0.1 µm to 2.1 ± 0.4 µm in 3%-gelatin, and 0.5 ± 0.1 µm to 1.6 ± 0.1 µm in 7%-gelatin, for excitation durations from 0.2 to 1 ms. The velocities were measured from 0.88 ± 0.27 m s^−1^ to 0.64 ± 0.04 m s^−1^ in 3%-gelatin, and 1.50 ± 0.30 m s^−1^ to 1.44 ± 0.19 m s^−1^ in 7%-gelatin. The results indicate that a longer excitation duration resulted in greater differences in the peak displacement between phantoms with different stiffnesses. In addition, the velocities remain almost identical when applying the excitation duration from 0.4 to 1 ms. As larger displacements and stable velocities were easy to detect and analyse, the excitation parameters were set as 170 V and 1 ms tone burst for the subsequent experiments involving the acquisition of high-resolution ARF elasticity images.

**Figure 8.**
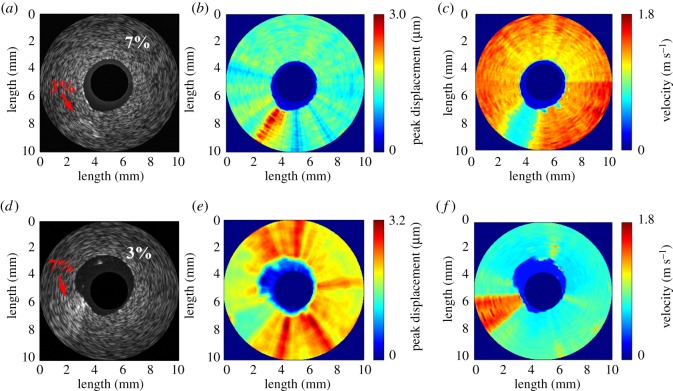
B-mode image (*a*), wave-amplitude image (*b*) and wave-velocity image (*c*) for phantom type I. B-mode image (*d*), wave-amplitude image (*e*) and wave-velocity image (*f*) for phantom type II.

**Figure 9.**
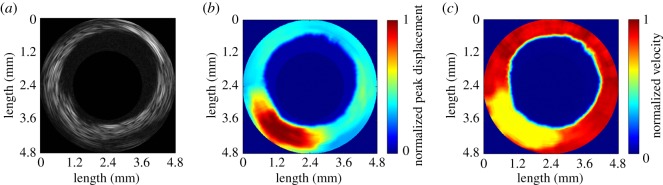
*Ex vivo* experiments for a rabbit aorta with a lipid-diet-induced plaque. B-mode image (*a*), wave-amplitude image (*b*) and wave-velocity image (*c*).

In the subsequent 2D ARF-IVUS elasticity imaging experiments (figures 8 and 9), the elasticity images were reconstructed based on the shear wave propagation induced by the ARF: the wave-amplitude images were formed from the peak displacements, while the wave-velocity images were formed from the shear wave velocities through equation (2.1). The pushing element was driven by 170 V with 1 ms tone bursts. Figure 8 shows the B-mode images with their corresponding wave-amplitude images and wave-velocity images for the gelatin phantoms—type I and type II. The dark-blue region in the figure indicates where no displacement or shear wave velocity occurred, which was in the saline solution and the region outside the field of view in both wave-amplitude and wave-velocity images. The darker region in the centre of the B-mode images in figure 8*a*,*d* corresponds to the region of the saline solution. For phantom type I, it is difficult to distinguish either the location or stiffness of the inclusion regions in the B-mode image shown in figure 8*a*. In contrast, the location and stiffness of the softer rod located between 7 and 8 o'clock are evident in the wave-velocity image in figure 8*c*. The velocities of the shear wave for the softer rod and the stiffer background were averaged to be 0.70 and 1.37 m s^−1^, respectively. On the other hand, the softer rod is located in the wave-amplitude image at around 7 o'clock in figure 8*b*. The peak displacements of the shear wave for the softer rod and the stiffer background were averaged to be 2.3 and 1.4 µm, respectively. For phantom type II, an inhomogeneity with bright speckles located between 8 and 9 o'clock in the B-mode image in figure 8*d* corresponds to the stiffer rod. Although the location of the stiffer rod is distinguished from the softer background in the B-mode image, this image does not provide any information about the stiffness distribution. In contrast, the stiffness distribution of the phantom is apparent in the wave-amplitude and wave-velocity images in figure 8*e* and *f*, respectively. The measured peak displacement and shear wave velocity were averaged to be 1.6 µm and 1.42 m s^−1^, respectively, for the stiffer rod, and 2.2 µm and 0.76 m s^−1^ for the softer background. The experimental results obtained from the phantoms verified that stiffness variations within the microstructure tissues can be detected by the ARF-IVUS elasticity imaging system.

Figure 9 shows the typical B-mode image and its corresponding wave-amplitude image and wave-velocity image for the rabbit aorta with a lipid-diet-induced plaque. The presence of the atherosclerotic lesion results in significant non-uniformity in the morphology in the B-mode image, as shown in figure 9*a*. While the use of high-frequency ultrasound to produce a conventional B-mode image is helpful for identifying the arterial structure, the speckle pattern cannot be directly related to the elastic properties [42]. In contrast, the distribution of stiffness in the atherosclerotic region can be observed in the wave-amplitude image and the wave-velocity image, as also shown in figure 9*b* and figure 9*c*, respectively. The softer region is located between 6 and 8 o'clock, for which the average peak displacement was 3.7 ± 1.2 µm and the average wave velocity was 0.38 ± 0.19 m s^−1^. However, the peak displacement and the velocity in the remaining region was averaged to be 1.0 ± 0.2 µm and 3.45 ± 0.45 m s^−1^, respectively. It is noted that the values of the elastographic map in figure 9 were normalized after log compression because of the large variation in each map.

## 4. Discussion

The intensities generated by the pushing element under different excitation parameters (e.g. excitation durations and excitation voltages) in the study are within the restrictions specified by the US Food and Drug Administration (FDA). The regulatory acoustic intensity from diagnostic ultrasound equipment is 430* *mW cm^−2^ for cardiac use according to the ‘Guidance for Industry and FDA Staff—Information for Manufacturers Seeking Marketing Clearance of Diagnostic Ultrasound Systems and Transducers' [43]. The acoustic intensity of IVUS imaging is included in cardiac applications in the regulation. The maximum acoustic intensity of 412.9 mW cm^−2^ used in the present study is lower than the regulatory intensity of 430* *mW cm^−2^. In other words, the maximum acoustic intensity used in this study for ARF-IVUS elasticity imaging is consistent with the regulatory limit specified by the FDA for IVUS imaging applications. This is also the reason why the maximum excitation parameter for this system is under 170 V with 1 ms tone burst.

The concept of combining ARF elasticity imaging and IVUS imaging for detecting and characterizing atherosclerotic plaques was recently realized by using the ARFI technique instead of the SWEI technique. To date, two ARFI-IVUS systems have been proposed. (i) Two modified commercial IVUS transducers (with central frequencies of 9 and 18 MHz) were used to generate an ARF in a programmable ultrasound imaging system. The system could generate sufficient radiation forces to induce displacements of 2.2 and 0.7 µm in a phantom with a Young's modulus of 3.0 kPa when using 9 and 18 MHz IVUS transducers, respectively [44]. (ii) Dual-frequency IVUS transducers of 6.5 MHz and 30 MHz were fabricated using stacked piezoelectric plates with two co-aligned beams. The lower frequency element of the transducer generated an ARF that induced displacements of approximately 1.5 µm in an 8.1 kPa phantom, and the higher frequency element measured the backscatter from the phantom [36]. Those studies demonstrated the feasibility of ARF-induced IVUS imaging, but data for biological tissue were not reported. Compared to those studies, our system could generate an ARF to induce displacements of 2.1 µm and 1.6 µm in phantoms with Young's moduli of 1.35 and 4.95 kPa (transformed from the values in shear moduli with a Poisson ratio of 0.5), respectively. As the pushing beam and the imaging beam in our system are not on the same axis, the detected displacements decreased due to the shear wave attenuation in the phantoms between the pushing and the imaging beam. However, the magnitudes of displacements are still large enough to image and analyse. The present study is also the first intravascular elastography based on the shear wave elasticity imaging technique.

Figure 7*a*,*b* demonstrates that the differences in velocity between the 3%- and 7%-gelatin phantoms were much larger than in the peak displacement responses for variable excitation times from 0.2 to 1 ms at an excitation voltage of 170 V. These results are consistent with previous studies finding that the temporal behaviour of tissue displacements induced by ARF is more useful than the spatial behaviour for distinguishing differences in the elastic tissues [38]. The speed (*c*_s_) of a shear wave propagating in a linear, isotropic, elastic material is related to the tissue shear modulus (*μ*) and the tissue density (*ρ*) according to

4.1 $$c_{s}=\sqrt{\frac{\mu }{\rho }}.$$

In this study, the shear wave velocities measured in the 3%- and 7%-gelatin phantoms were around 0.6 and 1.4 m s^−1^, respectively. Assuming that the density of the phantom is 1000 g cm^−3^, the calculated shear moduli of these two phantoms are 0.36 and 1.96 kPa, respectively, using equation (4.1). These results are consistent with the above-mentioned studies finding that the shear moduli of 3% and 7% gelatin were around 0.45 and 1.65 kPa, respectively [38,39]. The results also indicate that the system could be used to distinguish the elasticity differences of at least 1.6 kPa (shear modulus) between different tissues.

The shear wave velocity measured in the red region in figure 9*c* was on an average found to be 3.45 m s^−1^, which is consistent with previous reports of the shear wave velocity in arteries being typically in the range of 3 to 7 m s^−1^ [45–47]. However, when the shear wave propagates in a thin plate or a thin hollow cylinder surrounded by liquids, with the geometry close to that of an artery, the wave would be reflected by the boundaries of the medium, resulting in complex shear wave propagation, the so-called Lamb wave. As Lamb waves are highly dispersive, the elastic properties of the artery have been estimated by fitting the dispersion curve between the phase velocity and the frequency to an anti-symmetric Lamb wave model [29,48,49]. In the present study, the IVUS transducer was manufactured with two elements which were responsible for pushing and tracking the tissues simultaneously. As the distance between the two elements was fixed, this gave rise to difficulties in measuring the phase changes at several locations along the wave propagation direction. However, this limitation can be overcome by using an empirical analytical formula to approximate the phase velocity (*c*_p_) based on the group velocity (*c*_g_) at a certain frequency according to [29,50]

4.2 $${\text{c}}_{p}(\omega )=\sqrt{\frac{\omega hc_{g}}{2\sqrt{3}}},$$

where *h* is the thickness of the artery, *ω* is the angular frequency and *c*_g_ = *c*_s_ is the group velocity we measured. Therefore, the quantified shear modulus can be estimated by

4.3 $$\mu =\frac{12\rho {c_{s}}^{4}(\omega )}{h^{2}\omega^{2}}.$$

In the study, the thickness of the artery was measured to be about 1 mm, the central frequency of the generated wave spectrum was 1200 Hz and the group velocity of the arterial wall was averaged to be 3.45 m s^−1^, so its corresponding shear modulus was calculated to be 29.9 kPa through equation (4.3). According to the previous literature, the elasticity range of the artery is very large, spanning a few orders of magnitude from tens to hundreds of kPa, depending on many parameters such as testing conditions, species and, most importantly, measurement technique [48,51–54]. However, our results of the arterial elasticity are within the same order as the study using the Lamb wave model, which reported that the shear elasticity of porcine carotid artery was measured to be from 24.22 to 44.71 kPa [48].

A lipid-rich plaque is more dangerous than other types of plaques because it is more unstable and prone to rupture. A ruptured plaque could easily block the lumen of a small artery and cause thrombosis that leads to acute infarction and sudden coronary death [6,9,10]. A reliable index for predicting the rupture of a lipid-rich plaque would be strongly influenced by its viscoelastic properties [55]. The formation of a lipid-rich plaque is the essential mechanism in the early development of a rupture-prone plaque [56]. Previous studies have measured the shear modulus of lipid-rich plaques at around 0.07 to 0.3 kPa *in vitro* [57] and approximately 0.7 kPa *in vivo* [58]; these values are similar to those of the 3%-gelatin phantom in the present study. This close correspondence indicates that it is reasonable to use a gelatin phantom to represent a lipid-rich plaque when performing system verification. Moreover, in the rabbit atherosclerosis experiment, the average shear wave velocity in the softer region was measured to be 0.38 m s^−1^, as shown in figure 9*c*, and its corresponding shear modulus was calculated to be 0.06 kPa by using equation (4.3) with the measured central frequency of 330 Hz and thickness of 1 mm. The estimated value falls within the range of the elasticities of vulnerable lipid-rich plaques mentioned above [58].

In the phantom experiments in figure 8, artefacts were found in both wave-amplitude and wave-velocity images; the reason may be attributed to a viscous response from the gelatin gels. Viscosity can be considered as an additional and cumulative low-pass filtering effect and makes the temporal shape of displacement much broader, which distorts the image of the shear wave propagation [59,60]. Several groups further focused on the viscous issues: based on the viscoelastic inclusion simulation and experiment, Orescanin *et al*. indicated that the degree of distortion in elastographic image increases with viscosity [59]; the results from Bercoff *et al*. also demonstrated that the viscosity largely affects the shear wave shape in the near-field area compared with the far-field area [60]. These may be the reasons to induce artefacts and can cause the inclusion to be presented as a dispersive shape in the wave images in figure 8. Therefore, although the 3%-gelatin phantom has similar elastic properties to lipid-pool plaque, the viscous response to the plaques is different.

Although the elasticity of the artery and the plaque can be approximated by using the empirical analytical formula (equations (4.2) and (4.3)), it is still necessary to quantify the entire dispersion behaviour of the shear wave in order to completely characterize the mechanical properties of the artery and the plaque for obtaining more accurate mechanical properties (including elasticity and viscosity), and which will be the aim of our future work. Nevertheless, the original motivation for this work was to test a novel idea for applying ARF elasticity imaging to IVUS by using a dual-frequency IVUS transducer for the purpose of plaque and arterial wall characterization. The obtained results demonstrate the feasibility of the proposed ARF-IVUS elasticity imaging system that is based on using the peak displacement and velocity of the shear wave as simple metrics for distinguishing the arterial wall and a plaque.

The resolution of the present method of ARF-IVUS elasticity imaging could be further improved by using a detecting element operating at a higher frequency, such as a 60 to 120 MHz IVUS transducer, which is the highest frequency used in a multi-frequency IVUS application [61]. However, there is another issue to be resolved about the alignment of fore and aft elements of the transducer in this study. To reduce the higher interferences between the imaging and the pushing element, the distance between these two elements is maintained at about 0.5 mm. This means that the reconstructed shear wave velocity image is actually a hollow cylinder. Each of the estimated values of velocity might include several elastic components within the travelling trail of shear wave propagation. It might lead to an inaccurate estimation of elastic properties of the heterogeneous region in the plaque or the arterial wall. Therefore, a newly designed transducer aligned with more than two imaging elements spaced at a smaller distance is still necessary for *in vivo* applications in the future.

## 5. Conclusion

The feasibility of IVUS elasticity imaging based on ARF elasticity techniques was demonstrated in the study. A dual-frequency IVUS transducer with two elements was successfully used to induce shear wave propagation which could be simultaneously monitored. Suitable excitation parameters used to drive the pushing element of the system for producing the ARF have been found and measured within the restriction of FDA limitation. The phantom results demonstrated that the system can distinguish regions with different stiffnesses through the wave-amplitude and the wave-velocity images which were, respectively, reconstructed by measuring the peak displacement and the wave velocity of shear wave propagation. Moreover, stiffness distributions of the atherosclerotic aorta from the rabbit could be obtained from these elastographic images. All the results demonstrate that the ARF-IVUS elasticity imaging system has potential for further development towards clinical applications.

## Data accessibility

The datasets from this study are publically available on the Dryad Digital Repository (http://dx.doi.org/10.5061/dryad.h862n) [62].
